# Working Together to Support Self-Determination for Tāngata Kāpō (Blind and Low Vision) Māori: An Exemplar

**DOI:** 10.3390/ijerph21030343

**Published:** 2024-03-14

**Authors:** Bridgette Masters-Awatere, Rebekah Graham, Chrissie Cowan

**Affiliations:** 1 School of Psychology, University of Waikato, Hamilton 3216, New Zealand; bridgette.masters-awatere@waikato.ac.nz; 2 Parents of Vision Impaired New Zealand, Hamilton 3242, New Zealand; 3 Kāpō Māori Aotearoa New Zealand Inc., Hastings 4122, New Zealand; chrissie.cowan@kapomaori.org.nz

**Keywords:** Indigenous, relationship building, Kaupapa Māori, disability, blind, community psychology

## Abstract

This paper addresses the marginalisation of tāngata kāpō Māori (blind and low-vision Indigenous New Zealanders) in health- and vision-related research, despite New Zealand’s commitments to international conventions. Utilising a pūrākau-based approach, it challenges existing colonial narratives and emphasises the importance of Māori perspectives. We advocate for Māori self-determination over research processes. This paper shares insights from a systematic review and the development of a declaration for engaging with tāngata kāpō Māori, reflecting the 3-year collaborative process. The Materials and Methods section details a Kaupapa Māori-grounded data collection, prioritising relationships and cultural practices. Feedback loops with participants and forums ensure accurate representation. In conclusion, the study underscores NZ government obligations and presents the “3Rs” framework—relationships, respect, and reciprocity—as essential for meaningful research engagements with tāngata kāpō Māori. The findings contribute valuable insights to guide future research practices, advocating for the inclusion and recognition of tāngata kāpō Māori rights in practice and research.

## 1. Introduction

In Aotearoa New Zealand (NZ), disabled and Indigenous persons have the right to self-determination and to be treated in dignified and respectful ways. NZ is a signatory to several international conventions that set out the expectation and understanding of human rights for tāngata kāpō Māori (blind and low-vision Indigenous New Zealanders). International documents such as the United National Convention on the Rights of Persons with Disabilities (UNCRPD) and the United Nations Declaration on the Rights of Indigenous Persons (UNDRIP) complement He Whakaputanga o te Rangatiratanga o Niu Tireni (the Declaration of Independence of the United Tribes of New Zealand) of 1835 and Te Tiriti o Waitangi (The Treaty of Waitangi) of 1840 as key documents that uphold the rights of Māori, as Indigenous persons, to self-determination, partnership, and full participation in all echelons of NZ society. The NZ Disability Strategy [1] and the Māori Health Strategy [2] set out the government’s responsibilities (on behalf of the Crown) to disabled people and Māori in NZ.

Sadly, the rights of kāpō (blind, deafblind, low vision, vision impaired) to self-determination, partnership, and full participation are not always upheld within vision-related research. For example, researchers working with datasets that include Māori patients have not consistently connected their work with Māori knowledge or perspectives [3,4]. Within a disability context, Māori identities are poorly understood [5], and biomedical deficit-oriented models dominate research approaches [6]. Consequently, health and disability research tends to subsume the experiences of Māori into individualistic, colonial narratives [7].

Drawing on the works of Lee [8] and Pihama et al. [9], we take a pūrākau-based approach that counteracts settler–state narratives and works to decolonise health practice and research. Māori cosmologies and conceptualisations utilise storytelling (pūrākau) as a mechanism to communicate the wider cultural contexts within which people belong [10]. In this way, pūrākau connect understandings of disability and blindness to wider values, such as care for family members, inclusion, and the healthy expression of emotion. There has been a recent shift towards including Māori as a key stakeholder in vision-related research [11]. Nonetheless, the aspirations and priorities of those undertaking vision-related research are not necessarily aligned with tāngata kāpō Māori.

Our recent systematic review noted that vision-related research which drew on retrospective audits of pre-collected data consistently failed to consider the wider implications and contextual factors for Māori or for disabled persons [12]. Accessing de-identified data for retrospective analysis is a common practice in the field of medical health research. It is assumed that these existing datasets have robust, unproblematic, and correct details for Māori patients. However, this is not always the case [13,14]. Additionally, such studies do not routinely gain explicit consent by Māori for their medical data to be shared and analysed. Non-Māori who use decontextualised health data and who are oblivious to the wider sociocultural context can cause further harm to Māori [15]. As a result, Cormack and colleagues have advocated for a stronger Māori-led governance over health-related data to protect the interests of Māori [15,16].

Self-determination over health data and related research (data sovereignty) means Māori determine (either alone or jointly) the type of data collected, process of analyses, and the appropriate forms of reporting or dissemination [15]. This paper shares research learnings that have guided the development of ‘A declaration on engagement and exchange for tāngata kāpō Māori’ (See Appendix A), which is intended as a guide for other researchers who wish to engage with tāngata kāpō Māori.

## 2. Materials and Methods

The process for collecting data was collaboratively designed with representatives from two blind consumer groups: Kāpō Māori Aotearoa (CC) and Parents of Vision Impaired NZ (RG). Collective agreement amongst the team produced a research design grounded within Kaupapa Māori practice [9,17]. From the outset, this research prioritised relationships, engaged in specific processes of relationship building, and embedded appropriate cultural practices into research engagements [18]. Our research team comprised Māori (BMA, CC) and non-Māori (RG). We explicitly combined Kaupapa Māori with kāpōtanga (social practices and ways of being that are specific to kāpō) to ensure that interactions with participants, from the initial recruitment phone calls through to production of reports, were inclusive, accessible, and communicated value [19]. This approach was crucial to achieving our research aims and essential for creating the high level of trust necessary for the openness of sharing that occurred.

Our data collection engagements involved hosting research wānanga (learning discussion). These are distinct from Anglo-Saxon meetings [20], in that meeting protocols support an understanding of the tapu (sacredness) of learning [21]. Protocols that may be unfamiliar to non-Indigenous researchers, but are familiar to many Māori, include (while not being restricted to) opening speeches and karakia (ritual prayers), ensuring appropriate time to engage in whakawhanaungatanga (relationship building), answering questions of attendees, and the provision of manaakitanga (hospitality). Intertwined with these are ways of engaging that are specific to Māori, which are location- and context-specific, such as regular use of te reo Māori (Māori language), humour, care for others, and deference to kaumātua (older persons) [22]. Ethics approval was obtained from the University of Waikato Human Research Ethics Health Committee for each tranche of wānanga (reference: HREC(Health)2021#3; HREC(Health)2022#17). A paper detailing our methods and research approach is forthcoming.

Wānanga were held in three major cities across NZ’s two major islands and online via video conference. Depending on the number of attendees, the duration of wānanga was 2–5 h. Altogether, six research wānanga were held, four with tāngata kāpō Māori and whānau (*n* = 10, *n* = 8, *n* = 6, *n* = 7) and two with industry professionals (*n* = 2, *n* = 3). Wānanga participants ranged in age from young adult (18 years) to elderly (over 80 years). The vision ability of participants ranged from none to some, with a variety of vision conditions represented. Some participants met the criteria for legally blind, while others were whānau members of a blind child. To protect the anonymity of individuals and their whānau, all individuals have been given a pseudonym. Data gathered from wānanga were subjected to a general inductive analysis [23].

Alongside wānanga and inductive analysis, we engaged in iterative research discussions with tāngata kāpō Māori and whānau as subject matter experts. This activity occurred at each level of our work (direction, findings, discussion, dissemination), which collectively took place over 3.5 years. Engaging in iterative discussions kept our analytical and academic work centred on matters of value and importance to tāngata kāpō Māori; while the research team has strong links with the kāpō community, none of the research team are kāpō. Undertaking the research in this manner avoids replicating research by non-disabled and non-Māori people who publish work about disabled and/or Māori without appropriate engagement, consultation, or partnership [24,25]. In all instances, the analysis and final reports were first presented to tāngata kāpō Māori, and lengthy open and honest discussion was had, with feedback incorporated throughout. These processes of engagement ensured the research appropriately reflected kāpō experiences and priorities.

Regarding feedback loops, there were numerous opportunities. Initially, a summary analysis was presented to Ngā Rōpū o Manaaki Tangata Kotahitanga (MTK), a youth collective affiliated with Kāpō Māori Aotearoa (KMA). MTK members affirmed the accuracy of our summary and then presented it to the Minister for Disability Issues [26]. Feedback on our summary analysis then shaped a subsequent conference presentation and published paper [27]. The research team presented at the biennial KMA hui taumata national conference (in 2021 and 2023), to whānau Māori at the annual PVINZ national conference (in 2022 and 2023), and at an international Indigenous eye health conference (NATSIEH, 2023). At each forum, we described the project’s progress, presented overarching themes, and received feedback, which the team debriefed about and then added into the next phase of research activity. At each stage, we engaged relationally with tāngata kāpō Māori in governance and key leadership roles to discuss research directions and findings. Our process of repeatedly checking with participants, interested parties, and stakeholders reflects the process of member-checking described by Birt and colleagues [28]. Through our process of repeated engagements, which allowed for checking to confirm interpretation and representation, our team is confident that responses were captured appropriately and applied to the declaration.

## 3. Findings and Discussion

This section is presented in three core themes: firstly, relationships that are intentionally mutually beneficial and last beyond the research cycle; secondly, respect in the form of respectful engagements that acknowledge the cost of engagement; and thirdly, reciprocity as a foundational element. Each theme is discussed before presenting a set of clear statements to guide health professionals and vision researchers when seeking to engage with tāngata kāpō Māori.

### 3.1. Relationships

Across all our research engagements, there was a clear call for ongoing respectful and reciprocal relationships between health professionals, vision-related researchers, and tāngata kāpō Māori. Several participants shared stories from the not-too-distant past of being disrespected and of demeaning treatment. These past experiences left them feeling diffident about re-engaging and sensitised to deprecatory comments. In the following quote, Marie ponders assumptions regarding blindness, before sharing her feelings that reflect the comfort developed during the wānanga process:

I was just thinking before that if you don’t look like a blind person, but what does a blind person look like?, people put that stigma on you—“you’re not blind, you’re not wearing glasses.” That stigma that people put on others. I like this sort of kōrero. There’s not much more that I can add, our progress, you know, what we’ve learnt, what we’re learning… I like listening to your fellas kōrero, your educated kōrero [gesturing to the group].

Another discussion point was about the intersection of disability and being visibly Māori. Participants discussed their uncertainty regarding the reason for denigratory interactions and lackadaisical service. While participants did not always comment at the time, they were conscious of being treated differently. They pondered during the wānanga whether the barrier to services or their unpleasant experience was because they were blind or because they were Māori. Tipene exemplifies this in the below quote:

So do you wonder sometimes… even Kingi talked about this. Is it because I’m brown? Or because I’m disabled? The leaning is more because I’m brown, because in our [friendship] group, the two members that are more darker coloured, seemed to get a harder time, whereas the fairer members didn’t.

This uncertainty also extended to being disbelieved about having a vision impairment. Not all kāpō use a cane or have a guide dog. Sighted people often made assumptions and questioned the presence of a disability. Liam and Pearl (below) attended separate wānanga in different parts of the country, but provided comments that indicate similar experiences in this regard:

Liam: you’re already in a compromised position with eyesight. That perception if you don’t have the cane or any physical indicators… questioning, are you blind really? Questioning your condition without getting to know you.

Pearl: A worker snatched my cane off me and said, that’s only for disabled people. I stopped going… I had black sunglasses on… I went in [to the bank] and lined up. When I got up there, I said, “I’ve lost my card I need a new one”. She [bank teller] said “Take your glasses off”. I gave her my card. She said, “I want you to take your glasses off”. I was humiliated. So, I took them off and said, “I’m vision impaired.” I felt uncomfortable.

These quotes demonstrate that wānanga participants were highly aware of the differential treatment meted to members and made inferences related to the level of melatonin in their skin colour and to disability assumptions. The comments connect to the next core theme of our findings, that of being treated with respect.

### 3.2. Respect

Participants clearly articulated the expectation of being treated with respect, sharing stories of being disrespected when seeking assistance they were entitled to, across both health and social sectors. Lani comments below on the intersection of health and social support, and how respect in one area (with her GP) did not translate into needed supports:

It’s so annoying. Even my doctor tried to say something, but they [welfare service] said no… apparently, I don’t meet the criteria [for invalids’ benefit]? I’m vision impaired, I have a disability—what criteria do I have to meet?

Lani struggled with the ongoing disrespect she faced when attempting to access support from a government agency charged with providing support services. Such experiences left her reluctant to request assistance or to engage further, despite qualifying for support. Similarly, Matt comments below on the assumptions made from service providers:

In their head they think that everyone who walks through the door understands their system… people walk through the door [to the service], and they have no idea… you get treated a bit like you’re an idiot.

In this quote, Matt succinctly summarises the reality for many disabled—being treated “like an idiot”. As noted earlier, participants were never certain if such treatment was due to being disabled or being Māori. The systems, values, and practices that are embedded into both health and social support in NZ were initially based on the needs of able-bodied colonial persons. These values continue to permeate NZ’s health system today [29]. Aaliyah has reached an age in life (50+ years) where she now directly addresses stereotypes encountered. Here, she describes confrontation with a vision-related health professional:

Because of my age too, [people see me as] dumb… the specialist that did the [eye] test, I asked her, “Do I have dumb Māori tattooed on my face here [signals to forehead]” And she goes, “Oh no, I didn’t mean that,” and I said, “Yeah, that’s what you meant. That’s why you said what you said”.

Experiences of disrespect were affirmed as common amongst participants, with narratives recounted off-hand as an ordinary (everyday) occurrence across all wānanga held. To the research team, this common occurrence indicated the need to articulate respect as a core value preceding engagement.

Further discussions on the theme of respect related to governance, self-determination, and cultural lore. Reflections about COVID-19 restrictions centred on the place of mātauranga Māori (Māori knowledge that includes traditions, values, concepts, philosophies, world views, and understandings derived from uniquely Māori cultural points of view) as a more powerful driving force to live by, as opposed to Eurocentric law-based legalities. The following exchange between Kingi and Aaliyah exemplifies these discussions:

Kingi: It’s all white man’s law… Which is most powerful? Māori tikanga, or European law? And it’s always going to be Māori tikanga [cultural practices], because when you’re taught that from your tūpuna [ancestors], nothing is stronger than that.

Aaliyah: If we can, as the iwi groups were saying, we’re thinking about our whānau, our intergenerational health, in the first instance, why can’t you trust us to decide what is best for keeping our tikanga?

Kingi: In Māoridom, there’s two sides of the coin. One side is that you’ve got Anglo side. They’ve got the law on their side, but in Māori we have the Māori lore, and that’s all year round, 24/7, all around us. So when there’s a tangi you can’t break the Māori lore because the Māori lore makes you go… With the English law, [they] might lock you up, but they don’t understand what the Māori lore is because they never been in that area… In Māoridom the biggest thing for us is the Māori lore. The Māori lore. I don’t care who you are. You could be the king and the queen. If my loved one has passed away, that’s where my love goes to. Not to you, that’s the Māori lore.

In this exchange, Kingi and Aaliyah highlight the tensions of differing approaches, and allude to the disrespect often shown to Indigenous practices. This implicit disrespect for tikanga Māori (customary practices) plays out across multiple systems and interactions for individual Māori. Such collective experiences, over time, understandably result in distrust and reluctance to engage [6], which are further misinterpreted by non-Māori [24]. Undoing this harm takes time, ongoing respectful engagements, and an emphasis on ensuring consistency of high-quality experience.

### 3.3. Reciprocity

The final core theme is that of reciprocity. This refers to a range of practices that, when taken together, ensure an equitable exchange between tāngata kāpō Māori and service providers, health professionals, or vision researchers; reciprocity is not limited to the provision of vouchers after an interview. As several wānanga attendees comment below, reciprocity includes the sharing of ideas in a culturally supportive environment:

Mia: It’s interesting to share about the older generation. I definitely learnt a lot and I’m glad to be a part of the kōrero. I like the environment, being outside, it is nice.

Pearl: I enjoyed it [the wānanga], I enjoy this out here [referring to the cafe and outdoors table]. It’s really relaxed, we’re sharing kai (food) and that. It feels good. Thank you.

Liam: It’s [the wānanga] been really validating. This is what we needed with our conversations. We’re always doing our own things and making sure that what we’re doing to either improve or adapt or the use of something new like technology for instance to make us better equipped amongst society… we get real whakamā (shy/embarrassed) about trying to figure out if we’re doing the right thing or not, so it’s really nice to say what has or what hasn’t worked, particularly in this environment.

A sense of feeling heard and being listened to, of having their individual experiences validated through sharing with others, was promoted through the acts of reciprocity undertaken during the research. The iterative feedback cycles engaged also engendered a sense of wider value beyond their individual contributions. As Matt dryly asked at the first wānanga, “is [this research] more bureaucracy and lovely words and memos and nothing changes.” Similarly, Liam notes that dealing with ableism and racism through available channels, such as making a complaint, “takes extra work that I don’t have time for.” Comments shared about reciprocal relationships not leaving persons feeling discriminated against, but that their contributions to outputs will make positive changes for members of their community, are consistent with community research advice by Rappaport [30].

Both wānanga with health professionals and researchers produced valuable conversation on reciprocity and relational engagement. In particular, the way in which tertiary organisations and health research incentivises extractive research practices. The current system of research funding in NZ is heavily influenced by government agency funding priorities, whereby emphasis is on outputs rather than relational interactions that will benefit communities and disability groups. Emma comments on the challenges faced by new researchers below:

Structural issues to support research in Universities are perpetuated by funding rounds… Short-term research contracts make it difficult to engage in long-term research relationships.

Here, Emma articulates the difficulties that post-doctoral researchers in NZ must manage, that of insecure and uncertain research funding, and one where tenured employment is dependent on extractive methods that prioritize the researchers’ goals. Daniel, a health professional, expanded on Emma’s comment: “Existing rules in the medical system make it difficult to implement relational engagement.” Daniel gave an example from their practice of a patient who did not attend a scheduled appointment. Daniel’s initial preferred response was to walk down the road to where the patient lived to see if they were okay and to check why they did not attend. However, NZ practice required them to write to the patient’s GP, who would then contact the patient and request that they contact the clinic Daniel was working for. In this manner, the current medical system’s rules and principles are based on colonial ideals that favours objective distance from a professional. Daniel commented that it is useful to know what Māori perspectives are, so when non-Indigenous professionals are unsure, they have a guide to support relational engagement approaches. It became clear to us, the research team, that there was a need to craft a set of statements that include and build reciprocal engagements into the research relationship from the outset.

### 3.4. Declaration

Following the research wānanga, iterative discussions, and feedback loops engaged in, the research team drafted seven core statements which formed the basis of the Declaration on Engagement and Exchange (see Appendix A for more detailed text). These core statements provide a ‘road map’ for health professionals and vision researchers when seeking to engage with tāngata kāpō Māori. Presented by KMA, the declaration represents the voices of tāngata kāpō Māori, whose key instructions are as follows:

1. Engage from the beginning (at the time that the seed of an idea is cultivated) and then maintain throughout.
2. Resource accessibility appropriately. Make an initial plan and be mindful there are often additional costs for disabled people to travel, which should not be placed on disabled persons.
3. Commit to partnerships that produce a mutually beneficial arrangement. Partnership is a reciprocal arrangement. A contracted service is more appropriate for extractive approaches that upskill others but have no direct benefit for our people.
4. Come with a basic understanding of how to engage respectfully with Māori by reading prior and have basic te reo and tikanga.
5. Value disabled persons and their experiences.
6. Understand the limits of Western positivism and methods that inform your epistemological approaches to knowledge and being in the world.
7. We will not perpetuate deficit narratives and attitudes towards disabled people or Māori. Uphold the mana of our people.

Our findings align with that of published research in the field of health regarding engagement with Indigenous groups. Linda Tuihiwai Smith’s seminal work *Decolonising Methodologies* is well known in this regard [25]. Despite previous publications clearly stating the need to establish relationships [6,7], engage respectfully [18,19], and to ensure practices are reciprocal [9,17], our conversations with participants indicate that implementing these practices remains difficult to achieve. Through clearly stating a series of statements which underpin ways to engage with tāngata kāpō Māori, our findings can be drawn on by health professionals and vision researchers seeking to engage with Indigenous groups.

## 4. Conclusions

Earlier research has highlighted inconsistent connection to Māori knowledge and perspectives. Promises afforded by NZ’s declaration of independence and the Tiriti o Waitangi, as well as those proffered as a signatory to international conventions such as the UNCRPD and UNDRIP, the NZ government and its agencies (such as social and health services and research) have obligations to uphold the rights of Māori. Through a combination of hosting several wānanga and an iterative feedback process that took place over 3 years, our team co-produced a declaration that expressly communicates the expectations and understandings of tāngata kāpō Māori. Each statement reflects a particular dimension of the key themes of relationship, respect, and reciprocity that were discussed in forums described earlier in this paper. Collectively, these findings support our earlier research and emphasise that meaningful research engagements with kāpō Māori requires “3Rs”. The first R stands for relationships. Tāngata kāpō Māori acknowledge the importance of relationships that are intentionally mutually beneficial from the outset and continue throughout and beyond the cycle of funded research. The second R stands for respect. Respectful engagements appropriately acknowledge the cost of engagements and tāngata kāpō Māori expertise of their lived realities as well as that of other persons with living with disability. The third and final R stands for reciprocity. Participants affirmed that meaningful research interactions were founded on recognising reciprocity as essential and potentially energising for all involved. From here, having a clearly stated declaration to draw on, health professionals and vision researchers alike have the resources they need to engage in relational, respectful, and reciprocal practices.

## Data Availability

The full wānanga datasets and notes from discussions presented in this article are not publicly available due to supporting Māori data sovereignty practices.

## Appendix A. Kāpō Māori Aotearoa Declaration on Engagement and Exchange

“The gifts of kāpō”

**Opening mihi:** Karanga, karanga, karanga rā. Nau mai e te ata hapara e kawea mai te rangi hou! me maumahara tonu ki a rātou mā, e moe nei i te moenga roa, nō reira, moe mai, moe mai, moe mai rā. Ka rere tonu ra ngā kupu whakamihi ki tēnā, ki tēnā, ki tēnā o tātou ngā ringa raupaa e tautoko kaha nei ki tēnei kaupapa whakahirahira. Ma te whakaaro ko te korero, ma te korero ko te wānanga, ma te wānanga ka tau mai te matauranga hei oranga mā tātou katoa.

As we call to the dawning of a new day, we acknowledge those whom have gone before us and now are at rest in heavenly slumber. Rest on in love and peace. The words of gratitude and appreciation will forever flow to the many whom supported this significant collaboration! From thought comes language, from language comes learning, from learning comes knowledge for one and all!

**Declaration Intent:** To clearly identify ways in which individuals, groups, organisations, agencies, and governing bodies can approach and engage with KMA and its representatives in a way that upholds the tino rangatiratanga, kawanatanga and oretitanga of tāngata kāpō.

**Key documents:** This Declaration sits within a wider national and international context. Aotearoa New Zealand is a signatory to UNCRPD and UNDRIP. These international declarations promote and uphold the rights of disabled and Indigenous persons. Within Aotearoa New Zealand, He Whakaputanga o te Rangatiratanga o Niu Tireni and Te Tiriti o Waitangi are key documents upholding the rights of Māori to self-determination, partnership, and full participation in all echelons of society. The NZ Disability Strategy and the Māori Health Strategy clearly outline the Crown’s responsibilities to disabled and to Māori in Aotearoa NZ. Together, these documents uphold and protect the right of Māori to matauranga Māori which encompasses research epistemologies and tikanga Māori which upholds research methodologies. The expectation and understanding of human rights for tāngata kāpō Māori, as Indigenous persons, and upholds engagements and exchanges within this context.

**Declaration Core Statements:**

1. KMA and/or its representatives are there at the beginning, (at the time that the seed is cultivated) not just brought in at the end (at the time of harvest).
2. Resource yourself for accessibility costs. This includes having a basic working knowledge of access requirements for both tāngata kāpō and tāngata whaikaha, and an initial plan of how you might meet these requirements. Access requirements can be found online and quotes can be obtained from transport operators. There are often additional costs for disabled to travel (e.g., support persons, wheelchair hoist van) and these need to be built into your research budget not placed on the disabled person.
3. Come with an intent to partner with us. We are often happy to share our experiences within the context of a mutually beneficial arrangement. If you require upskilling in the areas of kāpōtanga or tikanga or mātauranga, and/or are seeking to extract information for your benefit, this is a consultancy arrangement and as such will need to be entered into contractually with the appropriate fee. A partnership is a reciprocal arrangement; a contracted service is more appropriate for extractive approaches that upskill others but with no direct benefit for our people.
4. Come with a basic understanding of how to engage respectfully with Māori. There are many resources available on working with Māori. We recommend you undertake some basic reading prior, have a little te reo, and a basic grasp of key tenets of tikanga.
5. Come with a value for disabled persons and our experiences. This means including recompense for our time and our knowledge.
6. Understand the limits of your epistemological approaches to knowledge and being in the world. Western positivism and methods have their limitations; how do you plan to bridge-build to te Ao Māori? There is a shared narrative across generations within te Ao Māori, and for this to be shared requires both individual and collective consent. The latter requires more time and preparation than an individual consent form; how will you obtain both individual and collective consent?
7. We will not be involved in any research or projects that perpetuate deficit narratives and attitudes towards disabled or Māori. We look for research and projects that change deficit narratives and which uphold the mana of our people.

## References

[1] Office of Disability Issues (2016). New Zealand Disability Strategy.

[2] Ministry of Health (2014). The Guide to He Korowai Oranga: Māori Health Strategy 2014.

[3] Ingham T.R., Jones B., Perry M., King P.T., Baker G., Hickey H., Pouwhare R., Nikora L.W. (2022). The Multidimensional Impacts of Inequities for Tāngata Whaikaha Māori (Indigenous Māori with Lived Experience of Disability) in Aotearoa, New Zealand. Int. J. Environ. Res. Public Health.

[4] Nikora L.W., Karapu R., Hickey H., Awekotuku N.T. (2007). Disabled Maori and Disability Support Options. A Report Prepared for the Ministry of Health.

[5] Hickey H., Kakoullis E.J., Johnson K. (2020). A Personal Reflection on Indigeneity, Colonisation and the CRPD. Recognising Human Rights in Different Cultural Contexts the United Nations Convention on the Rights of Persons with Disabilities (CRPD).

[6] Papaarangi R., Sarah-Jane P., Elana C., Rhys J., Anneka A., Esther W., Matire H. (2017). Achieving health equity in Aotearoa: Strengthening responsiveness to Māori in health research. N. Z. Med. J..

[7] Graham R., Masters-Awatere B. (2020). Experiences of Māori of Aotearoa New Zealand’s public health system. Aust. N. Z. J. Public Health.

[8] Lee J. (2009). Decolonising Māori narratives: Pūrākau as a method. MAI Rev..

[9] Pihama L., Southey K., Tiakiwai S. (2015). Kaupapa Rangahau: A Reader. A Collection of Readings from the Kaupapa Rangahau Workshop Series.

[10] Seed-Pihama J., Archibald J., Xiiem Q.Q., Bol J., Lee-Morgan J., De Santolo J. (2019). Naming our names and telling our stories. Decolonizing Research: Indigenous Storywork as Methodology.

[11] Burn H., Hamm L., Black J., Burnett A., Harwood M., Burton M.J., Evans J.R., Ramke J. (2021). Eye care delivery models to improve access to eye care for Indigenous peoples in high-income countries: A scoping review. BMJ Glob. Health.

[12] Graham R., Masters-Awatere B., Cowan C. (2023). What do we know about the intersection of being blind and being Māori in Aotearoa New Zealand? Taking an applied community psychology approach to a systematic review of the published literature. J. Community Appl. Soc. Psychol..

[13] Cormack D., McLeod M. (2010). Improving and Maintaining Quality in Ethnicity Data Collections in the Health and Disability Sector.

[14] Reid P., Cormack D., Paine S.J. (2019). Colonial histories, racism and health—The experience of Māori and Indigenous peoples. Public Health.

[15] Cormack D., Reid P., Kukutai T. (2019). Indigenous data and health: Critical approaches to ‘race’/ethnicity and Indigenous data governance. Public Health.

[16] Kukutai T., Cormack D., Wilson M., Kukutai T., Carroll S.R., Rodriguez-Lonebear D. (2020). ‘Pushing the space’: Data sovereignty and self-determination in Aotearoa NZ. Indigenous Data Sovereignty and Policy.

[17] Curtis E. (2016). Indigenous positioning in health research: The importance of Kaupapa Maori theory-informed practice. AlterNative.

[18] Cram F., McCreanor T., Smith L.T., Nairn R., Johnstone W. (2006). Kaupapa Māori Research and Pākehā Social Science: Epistemological Tensions in a Study of Māori Health. Hūlili Multidiscip. Res. Hawaii. Well-Being.

[19] Higgins N., Phillips H., Cowan C., Wakefield B., Tikao K. (2010). Growing up Kāpo Māori: Whānau, Identity, Cultural Well-Being and Health/E Tipu Kāpo Māori Nei: Whānaungatanga, Māramatanga, Māoritanga, Hauoratanga. Auckland. https://www.donaldbeasley.org.nz/assets/publications/haua-maori/Growing-up-kapo-Maori.pdf.

[20] Van E.W., Bufreeler C. (2015). Meetings All over the World. The Cambridge Handbook of Meeting Science.

[21] Mead H.M. (2003). Tikanga Māori: Living by Māori Values.

[22] Mahuika N., Mahuika R. (2020). Wānanga as a research methodology. AlterNative.

[23] Thomas D.R. (2006). A General Inductive Approach for Analyzing Qualitative Evaluation Data. Am. J. Eval..

[24] Hickey H., Wilson D. (2017). Whānau hauā: Reframing disability from an indigenous perspective. MAI J..

[25] Smith L.T. (2021). Decolonizing Methodologies: Research and Indigenous Peoples.

[26] Masters-Awatere B., Graham R., Cowan C. ‘Seeing’ Kāpō Māori: Making Visible the Experiences of Kāpō Māori during and after COVID-19. Summary analysis report presented to the Minister for Social Development, 15 June 2021. Wellington, NZ. https://researchcommons.waikato.ac.nz/handle/10289/14809.

[27] Masters-Awatere B., Cowan C., Graham R. (2021). ‘Seeing’ Kāpō Māori: Making Visible the Experiences of Kāpō Māori during and after COVID-19. Psychol. Aotearoa.

[28] Birt L., Scott S., Cavers D., Campbell C., Walter F. (2016). Member Checking: A Tool to Enhance Trustworthiness or Merely a Nod to Validation?. Qual. Health Res..

[29] King P.T. (2019). Māori with Lived Experience of Disability Part I. Wai 2575, #B22. Wellington, New Zealand. https://forms.justice.govt.nz/search/Documents/WT/wt_DOC_150437272/Wai%202575%2C%20B022.pdf.

[30] Rappaport J. (2016). Rethinking the Meaning of Research in Collaborative Relationships. Collab. Anthropol..

